# Impact of mothers’ health attitudes on dental health of their children

**DOI:** 10.1186/1878-5085-5-S1-A111

**Published:** 2014-02-11

**Authors:** Mare Saag, Jana Olak

**Affiliations:** 1 University of Tartu, Estonia

**Keywords:** dental caries, dental anxiety, prediction, prevention, children

## 

Dental caries is a common well-known multifactorial disease which affects most of the people. Parents can be the main factor in avoiding dental caries in their children. The level and the time of acquisition of mutans streptococci (MS) in infants is important, since caries risk in permanent dentition may be dependent on early colonization with MS. Parents’ behaviour can impact their children’s oral health habits. The dietary habits are very important: daily consumption of sugar containing drinks, especially during night, and frequent snacking are factors predisposing to the development of early childhood caries. Dental fear is one of the important factors influencing dental health of children. The relationship between dental anxiety and poor oral health is mediated by poor oral health habits and irregular dental visits. Maternal dental anxiety is strongly correlated with children’s anxiety. Mothers’ dental fear manifests as poor dental health and poor oral hygiene of themselves and their children. Dentally anxious mothers brushed their teeth and visited the dentist less frequently than non-anxious mothers. The child’s general fear of dentistry correlated with maternal (p=0.004) and paternal (p=0.005) dental fear. Comparing children’s dental health and fear of dentistry, it was found that dental health indices, like dmft/DMFT and dt/DT were strongly associated with children’s general fear of dentistry (p=0.017 and p=0.005, respectively), and with their fear of invasive (p=0.025 and p=0.001, respectively) and dt/DT with non-invasive procedures (p=0.004). Health education of mothers and simple preventive measures used by them reduce the risk of caries in their children. Xylitol candies, used by the mothers of toddlers, daily with adequate frequency, quantity and length of the time during eruption of primary teeth reduce the level of transmission of MS from mothers to their children. The reduced early colonization of children’s teeth with MS will manifest as improved dental health and reduced caries experience.

